# Evaluation of Factors Affecting Continuous Performance Test Identical Pairs Version Score of Schizophrenic Patients in a Japanese Clinical Sample

**DOI:** 10.1155/2012/970131

**Published:** 2012-04-02

**Authors:** Takayoshi Koide, Branko Aleksic, Tsutomu Kikuchi, Masahiro Banno, Kunihiro Kohmura, Yasunori Adachi, Naoko Kawano, Tetsuya Iidaka, Norio Ozaki

**Affiliations:** ^1^Department of Psychiatry, Nagoya University Graduate School of Medicine, 65 Tsurumai-cho, Showa-ku, Nagoya 466-8550, Japan; ^2^Department of Psychiatry, Matsuzaki Hospital, 67 Azamotosanbongi, Sanbongi-cho, Aichi, Toyohashi 441-8152, Japan

## Abstract

*Aim*. Cognitive impairment in schizophrenia strongly relates to social outcome and is a good candidate for endophenotypes. When we accurately measure drug efficacy or effects of genes or variants relevant to schizophrenia on cognitive impairment, clinical factors that can affect scores on cognitive tests, such as age and severity of symptoms, should be considered. To elucidate the effect of clinical factors, we conducted multiple regression analysis using scores of the Continuous Performance Test Identical Pairs Version (CPT-IP), which is often used to measure attention/vigilance in schizophrenia. *Methods*. We conducted the CPT-IP (4-4 digit) and examined clinical information (sex, age, education years, onset age, duration of illness, chlorpromazine-equivalent dose, and Positive and Negative Symptom Scale (PANSS) scores) in 126 schizophrenia patients in Japanese population. Multiple regression analysis was used to evaluate the effect of clinical factors. *Results*. Age, chlorpromazine-equivalent dose, and PANSS-negative symptom score were associated with mean d′ score in patients. These three clinical factors explained about 28% of the variance in mean d′ score. *Conclusions*. As conclusion, CPT-IP score in schizophrenia patients is influenced by age, chlorpromazine-equivalent dose and PANSS negative symptom score.

## 1. Introduction

Schizophrenia is a complex, heritable psychiatric disorder, affecting approximately 1% of the general population. The heritability of schizophrenia is estimated to be 64% [1]. Genes relevant to schizophrenia or variants that may modulate risk for the disease have been identified using both linkage and candidate-based or whole-genome association studies [2–5]. A complementary approach examines the genetics of schizophrenia from the neurobiological perspective with neurocognitive endophenotypic markers of putative brain function. The underlying brain dysfunctions (and related endophenotypes) are more stable, trait-like markers that can be used to refine the psychiatric diagnosis. This approach is further motivated by the need to elucidate pathophysiological pathways after candidate variants are established [6].

 The Consortium on the Genetics of Schizophrenia is a 7-site collaboration that examines the genetic architecture of quantitative endophenotypes in families with schizophrenia. The authors suggested that the Continuous Performance Test Identical Pairs Version (CPT-IP), Degraded Stimulus Continuous Performance Test (DS-CPT), Verbal Declarative Memory Test, Working Memory Test, and Penn Computerized Neurocognitive Battery are the most appropriate tests to evaluate endophenotypes relevant to schizophrenia. Furthermore, the heritability of attention/vigilance using sample comprised of 30 healthy families was estimated to be 0.39 and 0.49 based on verbal and spatial CPT-IP scores, respectively [7]. The effect size was 1.18 when schizophrenia patients and controls were compared. Accordingly, the comparison between the first-degree relatives of schizophrenic patients and controls resulted in smaller effect size (0.54) [6].

 CPT-IP is included as a core test in major psychological batteries used to evaluate cognitive functioning of psychiatric patients, such as the Measurement and Treatment Research to Improve Cognition in Schizophrenia and the Consensus Cognitive Battery (MCCB) for schizophrenia. Cognitive impairment is one of the core symptoms of schizophrenia and is associated with impaired quality of life and poor outcome [8–10]. The CPT-IP test used to evaluate one of the cognitive endophenotypes related to schizophrenia. Specifically, CPT-IP can measure the attention/vigilance deficit that is commonly found in schizophrenic subjects and those who are at risk for the disorder [11].

 Biological phenotypes (e.g., cognitive or central executive functions) are thought to more closely reflect the effects of genetic variation compared with manifested psychiatric illness; therefore, endophenotype studies have proven to be more robust and require smaller sample sizes than purely diagnosis-based studies. When genetic effects on cognitive performance are evaluated, it is important to consider measurement errors [12] as well as the effect of clinical factors that may strongly affect CPT-IP scores. In that regard, except for several reports that have evaluated the association between age and Positive and Negative Symptom Scale (PANSS) scores on cognitive performance [13], there are no comprehensive studies that looked for relevant covariates that may influence CPT-IP scores. We conducted an analysis of factors that can affect CPT-IP scores (e.g., sex, age, education years, onset age, duration of illness, chlorpromazine equivalent dose, and PANSS scores) using a Japanese population-based sample.

## 2. Methods

### 2.1. Participants

 This study was approved by the Ethics Committee of each participating institute, and written informed consent was obtained from each participant. Patients were included in the study if they (1) met DSM-IV criteria for schizophrenia, (2) were physically healthy, and (3) had no mood disorders, substance abuse, neurodevelopmental disorders, epilepsy, or known mental retardation. Consensus diagnoses were made by at least two experienced psychiatrists according to DSM-IV criteria on the basis of unstructured interviews with patients and families and review of medical records. The rate of the samples excluded due to a lack of consensus was less than 5%. All subjects were unrelated to each other, living in the central area of the mainland of Japan, and self-identified as Japanese. The study included 126 unrelated Japanese patients with schizophrenia (mean age, 44.4 ± 13.3 years; 80 males and 46 females).

### 2.2. Measurement Settings

There are a variety of CPTs, the more commonly used being CPT-X/AX, DS-CPT, and CPT-IP [11]. CPT-IP has evolved over the course of the New York High-Risk Project [14]. In CPT-IP, the target is defined as the second stimulus in any pair of identical stimuli. The benefit of using CPT-IP instead of the other tests is due to the structure and simplicity of the examination. In other words, no number or number sequence is specified, as in the X/AX design, and the subject does not need to memorize each stimulus presented as in the DS-CPT, which can increase the information-processing load. We used CPT-IP program Release 4.0 (NewCPT.exe, Copyright 1982–2004 by Barbara A. Cornblatt, All Rights Reserved). The PC monitor was 10.4′ and letter size was at least 2.2 × 1.5 cm [7]. The distance between the subjects and the monitor was at least 50 cm.

 Stimuli were flashed on the screen at a constant rate of 1 per set, with a stimulus “on” time of 50 msec and a stimulus “off” time of 950 msec. Stimuli were four-digit numbers and were presented 150 times. In each 150 trial conditions, 30 of the trials (20%) were target trials and required a response. Target trials were those on which the second of a pair of two identical stimuli appeared. Responses to target trials were scored as hits [7]. Condition also included a number of catch trials on which the stimulus presented was similar but not identical to that of the preceding trial. Responses to catch trials were considered a specific type of commission error, referred to as false alarms. There were 30 catch trials (20%) in our test. The remaining trials in both conditions were 90 randomly distributed fillers. Responses to filler trials, referred to as random errors, were also considered to be commission errors but were analysed independently of false alarms. We conducted the four-digit CPT-IP two times, with a resting time between the two examinations of 1 min. Mean d′ score was defined as the mean of first and second d′ score.

### 2.3. Clinical Factors

 Chlorpromazine (CPZ) equivalent dose was calculated according to standard methodology based on a Japanese clinical sample [15, 16]. The PANSS was used to evaluate the severity of symptoms in patients [17].

### 2.4. Statistical Analysis

IBM SPSS Statistics Version 19 was used for all analyses. Intraclass correlation coefficient was calculated in d′, hits, false alarm, and random errors. Multiple regression was performed for the analysis of mean of d′ score using clinical information (sex, age, education years, onset age, duration of illness, CPZ equivalent dose of antipsychotics, and PANSS score) (positive, negative and general psychopathology). Multiple regression models were analysed using forward-backward stepwise selection. Multiple correlation coefficient adjusted for the degree of freedom (*R*_*a*_^2^), analysis of variance (ANOVA) *P*-value, and Durbin-Watson ratio were calculated to evaluate the extent of model fitting. The significance level was set at *P* = 0.05. 

## 3. Results

Clinical profile of participants is shown in Table 1. The intraclass correlation coefficient (ICC) of the mean d′ score was 0.71 (Table 2). In multiple regression analysis, age, CPZ equivalent dose, and PANSS-negative symptom score were significantly associated with mean d′ score (Table 3). Durbin-Watson ratio indicated the absence of spurious regression. Although no strong correlation (>0.8) was observed in all clinical parameters, the Pearson's correlation between age and duration of illness was high (0.72).

## 4. Discussion

 CPT-IP is a major neurocognitive examination used to assess cognitive impairment among psychiatric patients. Included as a subtest in the MCCB, CPT-IP scores are often used to assess drug efficacy in clinical trials or endophenotypes in genetic studies. Confounding factors, such as measurement error or influence of clinical data, can hamper interpretation of results. Thus, to elucidate the effects of clinical data (age, sex, education years, duration of illness, onset age, CPZ equivalent dose, and PANSS score) on CPT-IP score in schizophrenia patients, we performed a multiple regression analysis in Japanese people suffering from schizophrenia.

### 4.1. Main Findings

 Age and PANSS-negative symptom score were statistically associated with mean d′ score in schizophrenia patients. This finding is in concordance with a previous study [18]. Additionally, our results suggest that CPZ-equivalent dose affects CPT-IP score. Overall, using a relatively large Japanese clinical sample of schizophrenia, we showed that age, CPZ-equivalent, dose and PANSS-negative symptom score can have a major effect on the CPT-IP scores and therefore should be taken into the account when interpreting results obtained from patients with schizophrenia. Age, CPZ-equivalent dose, and PANSS negative symptom score explained about 28% of the variance in mean d′ score.

### 4.2. Limitations

 There are several limitations that should be considered when interpreting the results of the present study. Multiple regression analysis findings in schizophrenia patients would benefit if we had been able to obtain more clinical information, such as IQ score and duration of untreated psychosis. As we could not find significant effects of sex, age at disease onset, duration of illness, and PANSS-positive and general psychopathology score in this study, weak effects of these factors might be observed when the sample size is increased. 

## 5. Conclusion

 We investigated how covariates (age, CPZ-equivalent dose, and PANSS-negative symptom score) affect mean d′ score of CPT-IP. This is the first study using a single independent large Japanese schizophrenia sample set, known as homogeneous in terms of genetic makeup. Our study suggested that those effects should be carefully considered especially when CPT-IP is performed to detect small effect size factors which are expected to be found in case of common risk variants associated with schizophrenia or cognitive-enhancing drugs. Thus, as CPT-IP is likely to be an endophenotypic measure in molecular genetic studies of schizophrenia in the post-genome-wide association study era [19, 20], our data show that careful assessment of confounding factors is essential for interpretation of findings.

## Figures and Tables

**Table 1:** Participants profile.

	Patients (*n* = 126)	
Sex		
Male	80	
Female	46	

	Mean	SD^a^

Age (y)	44.4	13.3
Education years (y)	12.4	2.4
Onset age (y)	26.7	10.0
Duration of illness (y)	17.6	13.0
Chlorpromazine equivalent dose (mg/day)	631.9	434.0
PANSS score		
Positive (7–49)	16.3	5.2
Negative (7–49)	19.0	5.5
General psychopathology (16–112)	36.2	9.3
Total (30–210)	71.6	17.7

Clinical diagnosis		
Paranoid type	46	
Disorganized type	3	
Catatonic type	1	
Residual type	65	
Unknown	11	

*Polytherapy*		
Antipsychotics		
Monotherapy	26	
Risperidone	62	
Olanzapine	16	
Aripiprazole	17	
Other atypical drug	3	
Typical drug	2	

^a^standard deviation.

**Table 2:** Measurement results of 4-digit CPT-IP.

P	Patients (*n* = 126)		
	Mean	SD^a^	ICC^b^
d′			
1st	1.29	0.84	0.71
2nd	1.55	0.96	
mean	1.42	0.84	

Hits (0–30)			
1st	18.4	7.2	0.77
2nd	19.6	6.9	

False alarms (0–30)			
1st	6.3	4.6	0.70
2nd	5.7	4.7	

Random errors (0–90)			
1st	4.8	8.9	0.53
2nd	3.5	4.9	

^a^standard deviation

^b^intraclass correlation coefficient.

**Table 3:** Multiple regression analysis of mean d′ score.

Multiple regression analysis						

Forward-backward stepwise selection						

Clinical factors	Patients (*n* = 126)					
	Setting: *P*_in_ = 0.05, *P*_out_ = 0.1					
	PRC^a^	S-PRC^b^	VIF^c^	95% CI^d^		*P* value
				Lower	Upper	
Age (y)	−0.031	−0.45	1.04	−0.041	−0.020	<**0.001**
CPZ-equivalent dose (mg/day)	−0.00038	−0.21	1.01	−0.001	<0	**0.012**
PANSS-negative symptom score (7–49)	−0.026	−0.16	1.04	−0.052	<0	**0.017**
Intercept	3.54	—	—	2.89	4.19	<0.001

${\hat{R}}^{2\text{ e}}$	0.28					
ANOVA *P* value	<**0.001**					
Durbin-Watson ratio	1.93					

^a^partial regression coefficient

^b^standardized partial regression coefficient

^c^variance inflation factor

^d^confidence interval

^e^multiple correlation coefficient adjusted for the degrees of freedom.

## References

[1] Lichtenstein P, Yip BH, Björk C (2009). Common genetic determinants of schizophrenia and bipolar disorder in Swedish families: a population-based study. *The Lancet*.

[2] Stefansson H, Ophoff RA, Steinberg S (2009). Common variants conferring risk of schizophrenia. *Nature*.

[3] Shi J, Levinson DF, Duan J (2009). Common variants on chromosome 6p22.1 are associated with schizophrenia. *Nature*.

[4] Purcell SM, Wray NR, Stone JL (2009). Common polygenic variation contributes to risk of schizophrenia and bipolar disorder. *Nature*.

[5] Ng MYM, Levinson DF, Faraone SV (2009). Meta-analysis of 32 genome-wide linkage studies of schizophrenia. *Molecular Psychiatry*.

[6] Gur RE, Calkins ME, Gur RC (2007). The consortium on the genetics of schizophrenia: neurocognitive endophenotypes. *Schizophrenia Bulletin*.

[7] Cornblatt BA, Risch NJ, Faris G, Friedman D, Erlenmeyer-Kimling L (1988). The continuous performance test, identical pairs version (CPT-IP): I. New findings about sustained attention in normal families. *Psychiatry Research*.

[8] Green MF (1996). What are the functional consequences of neurocognitive deficits in schizophrenia?. *American Journal of Psychiatry*.

[9] Green MF, Kern RS, Braff DL, Mintz J (2000). Neurocognitive deficits and functional outcome in schizophrenia: are we measuring the “right stuff”?. *Schizophrenia Bulletin*.

[10] Green MF, Kern RS, Heaton RK (2004). Longitudinal studies of cognition and functional outcome in schizophrenia: implications for MATRICS. *Schizophrenia Research*.

[11] Cornblatt BA, Keilp JG (1994). Impaired attention, genetics, and the pathophysiology of schizophrenia. *Schizophrenia Bulletin*.

[12] Kendler KS, Neale MC (2010). Endophenotype: a conceptual analysis. *Molecular Psychiatry*.

[13] Nuechterlein KH, Green MF, Kern RS (2008). The MATRICS consensus cognitive battery, part 1: test selection, reliability, and validity. *American Journal of Psychiatry*.

[14] Erlenmeyer-Kimling L, Cornblatt BA (1992). A summary of attentional findings in the New York high-risk project. *Journal of Psychiatric Research*.

[15] Inagaki A, Inada T (2008). Dose equivalence of psychotropic drugs. Part XX: dose equivalence of novel antipsychotics: blonanserin. *Japanese Journal of Clinical Psychopharmacology*.

[16] Inagaki A, Inada T (2010). Dose equivalence of psychotropic drugs. Part XXII: dose equivalence of depot antipsychotics III: risperidon long-acting injection. *Japanese Journal of Clinical Psychopharmacology*.

[17] Kay SR, Fiszbein A, Opler LA (1987). The positive and negative syndrome scale (PANSS) for schizophrenia. *Schizophrenia Bulletin*.

[18] Nieuwenstein MR, Aleman A, De Haan EHF (2001). Relationship between symptom dimensions and neurocognitive functioning in schizophrenia: a meta-analysis of WCST and CPT studies. *Journal of Psychiatric Research*.

[19] Burdick KE, DeRosse P, Kane JM, Lencz T, Malhotra AK (2010). Association of genetic variation in the MET proto-oncogene with schizophrenia and general cognitive ability. *American Journal of Psychiatry*.

[20] Cannon TD (2010). Candidate gene studies in the GWAS era: the MET proto-oncogene, neurocognition, and schizophrenia. *American Journal of Psychiatry*.

